# Use of Mechanical Versus Biological Aortic Valve in Aortic Valve Replacement Surgery for Patients Aged 60–70 Years: A Systematic Review and Meta-Analysis

**DOI:** 10.7759/cureus.93571

**Published:** 2025-09-30

**Authors:** Nabila Natasya, Joseph Galea, Hannah Khan, Yugendranath Pagolu

**Affiliations:** 1 Emergency Department, South Tyneside and Sunderland NHS Foundation Trust, South Shields, GBR; 2 Surgical Science, University of Edinburgh, Edinburgh, GBR; 3 Cardiac Surgery, Mater Dei Hospital, Msida, MLT

**Keywords:** aortic valve replacement, cardiac surgery, elderly patients, meta-analysis, mortality

## Abstract

One of the cardiac surgical procedures that is performed in a steady number over time in the United Kingdom (UK) is aortic valve replacement (AVR) surgery, especially in those aged 60-70 years old.There has been various debates regarding which valve has better outcome in this group, and no guidelines have fixed recommendation for it. This research aims to examine the effects of AVR surgeries involving mechanical and bioprosthetic valves in individuals between the ages of 60 and 70. This metanalysis study was conducted by following the Preferred Reporting Items for Systematic Reviews and Meta-Analyses (PRISMA) guidelines, using secondary data from previous published studies. Outcomes include mortality, re-operation, thromboembolic event, and bleeding event. Inclusion criteria include direct comparison of using mechanical and biological valves, involve patients aged 60-70 years, and provide sufficient details of hazard ratio (HR), odds ratio (OR), and Kaplan-Meier curves. Collected data were analyzed using IBM SPSS Statistics version 29.0. We found that there Is a significant protective effect of mechanical valve on mortality compared to bioprosthetic valve (p = 0.00) and reoperation (p = 0.00). We conclude that mechanical valve emerges as a favorable option for SAVR in patients aged 60-70 years.

## Introduction and background

Aortic stenosis (AS), a common valvular disorder in older adults, can be congenital or acquired. Congenital AS is most often due to a bicuspid aortic valve, particularly in patients under 70 in developed countries, and is frequently complicated by calcification [1]. Acquired AS, more common in developing regions, is primarily caused by rheumatic disease, while other causes include tricuspid valve calcification, metabolic and systemic disorders, prior chest irradiation, and mineral metabolism abnormalities, such as those found in end-stage renal disease [1,2]. AS may obstruct left ventricular outflow at, above, or below the valve, as seen in hypertrophic cardiomyopathy [1]. Clinically, patients present with exertional dyspnea, chest pain, syncope, and eventually heart failure, with an earlier onset in bicuspid valves. Severe disease can lead to pulmonary congestion, angina, and reduced exercise tolerance. Syncope may arise from impaired cerebral perfusion, and complications include gastrointestinal bleeding and cerebral emboli. On examination, a delayed, weak carotid pulse and a mid-systolic ejection murmur, sometimes radiating to the apex, are characteristic findings [2,3].

Treatment for severe AS mostly involves aortic valve replacement (AVR) surgery [4,5]. Severe AS has a negative influence on health outcomes; therefore, it requires complex treatment techniques to reduce symptoms and lower risks. From these approaches, surgery, specifically, AVR, stands out as a vital treatment option that can help patients meet their goals by relieving symptoms and improving their prognosis. Research indicates that 5% of the global population has AS, and 50% of severe cases may result in death within two years if treatment is not received [5]. 

An increasing number of cardiac surgical operations have been carried out in the UK throughout time, including surgical aortic valve replacement (SAVR). Data from the National Cardiac Audit Programme in the UK shows that around 5,000 isolated first-time SAVR procedures are performed every year in the UK from 2014 to 2020. In the UK, patients aged 60-69 years are the ones who received cardiac surgery the most compared to other age groups with 7,760 patients undergoing cardiac surgery over the period of 2013-2021 [6]. This population also has the highest percentage of SAVR performed in the UK, with 2,752 procedures performed during the same period [6]. Although the number of SAVRs performed dropped significantly in 2020 onwards due to an increase of transcatheter aortic valve implantation (TAVI) procedures [6], the older population still needs open surgery. A study shows that 1.48% of the UK population aged 55 years and older in 2019 are affected by severe AS, with more patients indicated for SAVR compared to TAVI (116.000 vs. 51.000) [7]. 

Based on the European Society of Cardiology (ESC)/European Association for Cardio-Thoracic Surgery (EACTS) guidelines established in 2021, the eligibility criteria for SAVR patient candidates include severe aortic stenosis symptoms, a left ventricular ejection fraction (LVEF) below 50%, being under 75 years old, and being deemed low risk for SAVR based on the EuroScore II [4]. In addition, those who are unsuitable candidates for TAVI would also be eligible for SAVR. Severe aortic stenosis is characterized by specific hemodynamic parameters such as a mean gradient exceeding 40 mmHg, a peak velocity surpassing 4.0 m/s, and an aortic valve area less than 1.0 cm^2^, or by the presence of low-flow, low-gradient with reduced EF and flow reserve as observed in dobutamine stress echocardiography. In addition to AS cases, SAVR is also indicated in patients with symptomatic aortic regurgitation (AR) with significant enlargement of the ascending aorta, or asymptomatic but having left ventricular systolic dysfunction (LVSD) >50 mm and resting LVEF of 55% [4].

In the National Institute for Health and Care Excellence (NICE) guidelines, patients experiencing symptomatic severe aortic stenosis necessitate intervention. The EuroSCORE II is utilized as a reference for the intervention selection procedure. Patients with minimal surgical risk (less than 4%) or moderate risk (between 4% and 8%) are considered to be suitable candidates for SAVR, given that TAVI is not cost-effective for those patients. Furthermore, those who are determined unfit for TAVI because of challenges such as improper vascular access for the insertion of the TAVI catheter, an incorrect valve, an unusual shape of the aortic root and ascending aorta, or a notable calcium distribution in the aorta, are recommended for SAVR [8]. Patients with asymptomatic AR who have LVEF <55% or end-systolic diameter (ESD) >50 mm or end-systolic diameter index (ESDI) >24 mm/m^2^ on echocardiography are also recommended for SAVR [8].

The type of valve recommended in SAVR, especially for patients aged 60-70 years old, has not been established yet in any guidelines. According to the ESC/EACTS guidelines, a mechanical valve is preferable for those younger than 60, while a bioprosthetic valve is endorsed for patients older than 65 years. However, for those between 60 and 70, both valves can be used [9]. Meanwhile, according to the American Heart Association (AHA) guideline, for those who are aged less than 60 years, a mechanical valve is recommended, and for those who are older than 70 years, a bioprosthetic valve is indicated. This guideline also stated that both valves can be used for those aged 60-70 years [10].

There has been a debate about which type of valve is more suitable for this age group. A Swedish study using a database from 1997 to 2013 discovered a superiority of mechanical valve in terms of long-term survival among patients aged 50-69, although they are more at risk for major bleeding when compared to bioprosthetic valves [11]. A meta-analysis study comparing the use of bioprosthetic and mechanical valves among patients aged 50-70 also shows that the mechanical valve has better long-term survival [12]. Nonetheless, another study also found that there is no significant difference in using either valve in patients older than 60 years [13]. Moreover, there is a higher risk of bleeding when using mechanical valves in patients older than 60 years compared to bioprosthetic valves due to the difficulty in controlling the therapeutic level of warfarin among patients receiving mechanical valves [14]. The difficulty in deciding which type of valve to use for AVR in patients aged 60-70 makes it important for us to analyse this issue, since this age group is the one that statistically receives the most AVRs. This research aims to analyse the benefits and disadvantages of using a mechanical compared to a bioprosthetic valve in AVR procedure among patients aged 60-70 years.

## Review

Methods

This systematic review with meta-analysis was conducted by following the Preferred Reporting Items for Systematic Reviews and Meta-Analysis (PRISMA) 2020 guideline statement and checklist and has been registered with the University of Edinburgh Surgical Sciences Ethics Review Committee. This study was based on a protocol that predefined search strategy, selection criteria, outcomes, and statistical analysis plan. 

Selection Criteria and Search Strategy

Our systematic review adopted a broad inclusion criterion, encompassing randomised controlled trials (RCTs) and observational studies (OS) published in English across all years. We specifically targeted studies that provided a direct comparison between different mechanical and bioprosthetic valves in SAVR. These selected studies were required to have a minimum follow-up period of one year and to assess at least one of the outcomes of interest. Furthermore, our inclusion criteria were tailored to focus on studies involving patients aged 60-70 years, as this demographic represents a crucial subset of the population undergoing AVR surgery. Studies need to provide at least one of the following outcomes: all-cause mortality, re-operation, thromboembolic events, or bleeding events. In addition, we emphasized the importance of studies that provided detailed data on hazards ratio (HR), odds ratio (OR), and Kaplan-Meier curves, which are essential for a comprehensive analysis of outcomes. From January to February 2024, an extensive electronic literature search was conducted using Medline, PubMed, Web of Science, Cochrane Library, and Google Scholar. The search used terms that we included can be seen in Table 1. Studies that focus on TAVI, case reports, reviews, editorials, or conference abstracts without full-text data, duplicated populations, and not providing extractable outcomes are excluded from this study. 

**Table 1:** Search terms

"Aortic valve replacement" OR "AVR" OR "aortic valve surgery” AND "elderly" OR "geriatric" OR “aged"
AND
"Mechanical valve “OR "artificial valve" OR "type of valve" OR "valve choice" OR "valve preferences”
AND
"Bioprosthetic heart valve" OR "xenograft valve" OR "biological valve

The combinations between those words are also used as keywords or MeSH terms in the studies' titles or abstracts. In addition, the reference lists of salvaged articles were manually investigated for potentially related studies. In cases where study populations were duplicated across different publications, the study with the lengthiest follow-up period was chosen.

Data Extraction

The process of conducting this systematic review commenced with the extraction of search results from various databases, which were subsequently input into Covidence, a specialised software for systematic reviews. Through meticulous screening, relevant studies were identified, and full-text publications were procured for comprehensive examination. Data extraction was conducted meticulously, encompassing a range of critical elements sourced from article texts, tables, and figures. These included primary author details, publication year, study period, duration of follow-up, population characteristics, study design, the number of subjects undergoing AVR receiving biological or mechanical valves, and the outcomes of interest. The primary focus of this review rested on key outcomes such as the composite incidence of mortality and re-operation rates. Mortality was described as death from any cause occurring during the follow-up period, while re-operation encompassed instances where patients necessitated repeat surgery for AVR. Secondary endpoints encompassed bleeding and other valve-related complications, such as thrombosis, defined according to predefined criteria outlined in the included studies. Data pertaining to outcomes were meticulously extracted from a 10-year follow-up period of each study. This information was sourced from readily available data, including tables, graphs, and text narrations within the publications, as well as supplementary data. In instances where raw data were not directly accessible, efforts were made to contact the primary authors via email to obtain the necessary information. This rigorous approach to data extraction ensured the comprehensive acquisition of relevant information essential for the robust analysis and synthesis of findings in this systematic review.

Quality Assessment

The evaluation of bias in RCTs employed the assessment tools outlined in the Cochrane Handbook of Systematic Review of Interventions. Various factors were scrutinized, including sequence generation, allocation concealment, blinding, incomplete outcome data, selective outcome reporting, and other potential sources of bias. For the quality assessment of the included observational studies, the Newcastle-Ottawa Quality Assessment Scale (NOS Scale) was utilised. This assessment system for quality involved the examination of cohort studies from three key perspectives: the selection of study groups, the comparability of the groups, and the ascertainment of the outcome of interest.

Statistical Analysis

The statistical analysis conducted for this study utilized IBM SPSS Statistics for Windows, version 29.0 (released 2022, IBM Corp., Armonk, NY) [15]. Baseline demographics were scrutinized using the Chi-squared test for dichotomous variables and the T-test for continuous variables. A random effects model was employed to evaluate the impact of mechanical versus biological valves on clinical outcomes during follow-up, presented as the OR calculated on the logarithmic scale using generic inverse variance. A statistically significant overall effect was determined by a P value less than 0.05 or when the 95% confidence intervals did not overlap the line of no effect on forest plots. Heterogeneity among the involved studies was assessed using the Cochrane Q test, with the I2 statistic indicating the percentage of total variation across studies. Moderate heterogeneity was considered present at 50% or higher, while values exceeding 75% signified high heterogeneity. To visually evaluate potential publication bias, funnel plots were utilized. In addition, Egger's weighted regression statistic was applied to detect significant publication bias, with a P value <0.05 indicating its presence. These meticulous statistical analyses were crucial for elucidating the nuanced relationships between valve types and clinical outcomes, providing valuable insights for clinical decision-making in the field of cardiac surgery.

Results

Study Selection

The initial phase of our systematic review involved a comprehensive search strategy across multiple online databases, yielding a total of 652 articles identified through the selected keywords. Specifically, this included 196 articles from Medline, 172 from PubMed, 160 from Web of Science, 84 from Google Scholar, and 40 from ClinicalTrials.gov. To ensure the integrity of the data, these articles were imported into Covidence, a specialized software tool, for duplicate removal, resulting in the elimination of 165 redundant articles. Subsequently, articles were meticulously screened based on predetermined eligibility criteria, leading to the exclusion of 175 articles that did not meet the specified study design criteria, focusing primarily on RCTs or cohort studies. Following this initial screening, titles and abstracts were scrutinized to further exclude studies deemed irrelevant to the study question, narrowing down the pool of potentially relevant articles. Further refinement was achieved through manual assessment of the full texts of remaining articles, resulting in the identification of seven studies that met the stringent inclusion and exclusion criteria. Among these, one RCT and six observational cohort studies were deemed suitable for inclusion in our systematic review. Articles were excluded from consideration for various reasons, including incomplete data, utilisation of transcatheter approaches, non-English publication, dissimilar outcomes or comparators, and failure to include populations pertinent to our study objectives. Moreover, a thorough manual search of the bibliographies of selected articles yielded no additional relevant studies.

**Figure 1:**
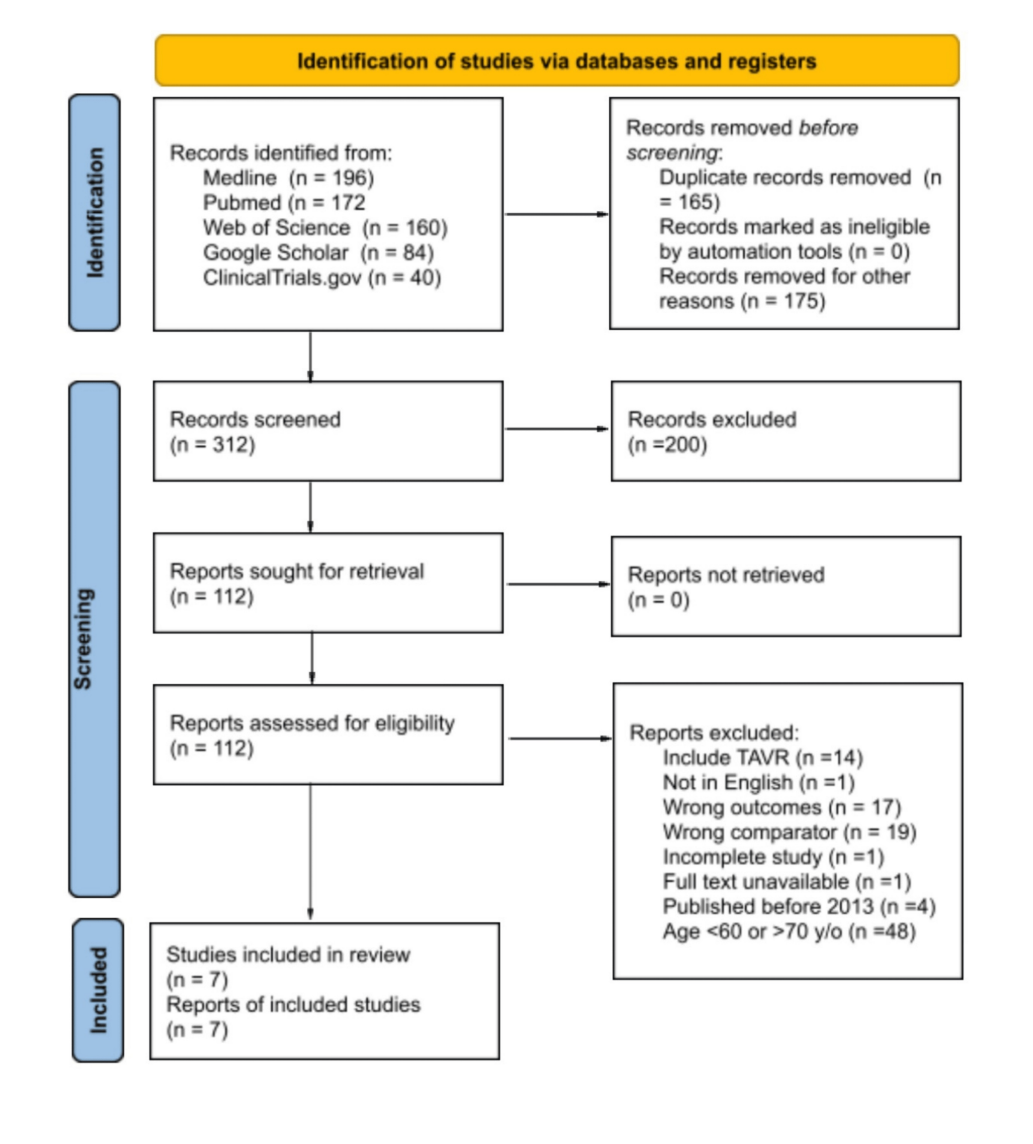
Preferred Reporting Items for Systematic Reviews and Meta-Analyses (PRISMA) flowchart for search strategy

Study Characteristics

A total of 15,189 patients who underwent SAVR, as documented across one RCT and six retrospective cohort studies. The average follow-up period across these studies was reported to be 14.14 ± 3.2 years. Among the patient cohort, 9,156 individuals received mechanical aortic valves, while 5,924 were implanted with bioprosthetic aortic valves, indicative of a substantial sample size for analysis. The comprehensive characteristics of the included studies are accurately summarized in Table 2. 

**Table 2:** Characteristics of studies included in the systematic review. MV: mechanical valve; BV: bioprosthetic valve; PSM: propensity score matching; M: mortality; RO: re-operation; B: bleeding; T/S: thromboembolic events/ stroke

Authors	Study type	Data collection period	Country	Type of valve used	MV	BV	Follow-up period	Reported outcomes			
								M	RO	B	T/S
Glaser et al., 2016 [11]	Multi-centre cohort retrospective + PSM	1997-2013	Sweden	Not mentioned	751	751	15 years	V	-	-	-
Nishida et al., 2014 [16]	RCT	1981-2013	Japan	M: CarboMedics; St. Jude, ATS valve B: Toronto Stentless Porcine Valve and 25 Medtronic Freestyle, Mitroflow BP	128	63	20 years	V	V	V	V
Sakamoto et al., 2016 [17]	Single-centre cohort retrospective	1995-2014	Japan	M: St. Jude Standard, St. Jude Regent, ATS, and CarboMedics. B: Carpentier-Edwards Perimount, Magna, Medtronic Mosaic, and St. Jude	41	83	15 years	V	V	V	V
Buckley, 2020 [18]	Single-centre cohort retrospective + PSM	2006-2014	Canada	Not mentioned	56	49	12 years	V	-	-	-
Zhao et al., 2023 [19]	Single-centre cohort retrospective	2002-2007	China	M: St. Jude, ATS, Medtronic, Sorin, Carbo. B: Carpentier-Edwards Perimount, Hancock, Hancock II, and Mosaic valves	101	87	15 years	V	-	V	V
Brennan et al., 2013 [20]	Multi-centre retrospective cohort	1991-1999	USA	Not mentioned	5949	3505	12 years	V	-	-	-
Minakata, et al., 2017 [21]	Multi-centre cohort retrospective + PSM	1985-2001	Japan	M: CarboMedics; St. Jude, ATS valve. B: Toronto Stentless Porcine Valve and 25 Medtronic Freestyle, Mitroflow BP	92	220	10 years	V	V	V	V

Baseline Demographics

The study cohort comprised predominantly male patients, accounting for 63.7% of the total population, corresponding to 5,232 individuals. To elucidate any potential disparities in demographic characteristics between recipients of mechanical and bioprosthetic valves, chi-square tests were conducted, revealing no statistically significant differences. Further insights into the demographic comparisons are provided in Table 3.

**Table 3:** Baseline demographics AF: atrial fibrillation; HF: heart failure; CAD: coronary arterial disease; MV: mechanical valve; BV: bioprosthetic valve

Variable	Studies (n)	Size (n)	Prevalence N,(%)	MV N,(%)	BV N,(%)	P-value
Male	4	5232	3335 (63.7)	1784 (61.6)	1352 (63.3)	0.118
AF	3	5108	432 (8.5)	269 (5.3)	162 (3.2)	0.114
HF	3	4774	682 (14.3)	395 (8.3)	287 (6.0)	0.291
Diabetes	4	5232	655 (12.5)	312 (6.0)	343 (6.6)	0.588
Renal disease	3	4774	333 (13.3)	331 (6.9)	302 (6.3)	1.178
CAD	4	5232	413 (7.9)	213 (4.1)	200 (3.8)	0.118

Clinical Outcomes

All seven included studies reported mortality outcomes in 15,189 patients [11,16-21]. A total of 5,698 patients were included in the period of 10 years after the surgery. Random model of meta-analysis shows that there is a meaningful negative effect of mechanical valve on mortality compared to bioprosthetic valve (OR 0.77; 95%CI 0.67-0.90; p = 0.00). This means that there is approximately a 23% risk of mortality reduction in using a mechanical valve among patients aged 60-70 years who undergo SAVR. A heterogeneity test was performed, and it was found that there was low heterogeneity present between studies (I2 = 21%), but there was no homogeneity detected (p = 0.51). 

**Figure 2:**
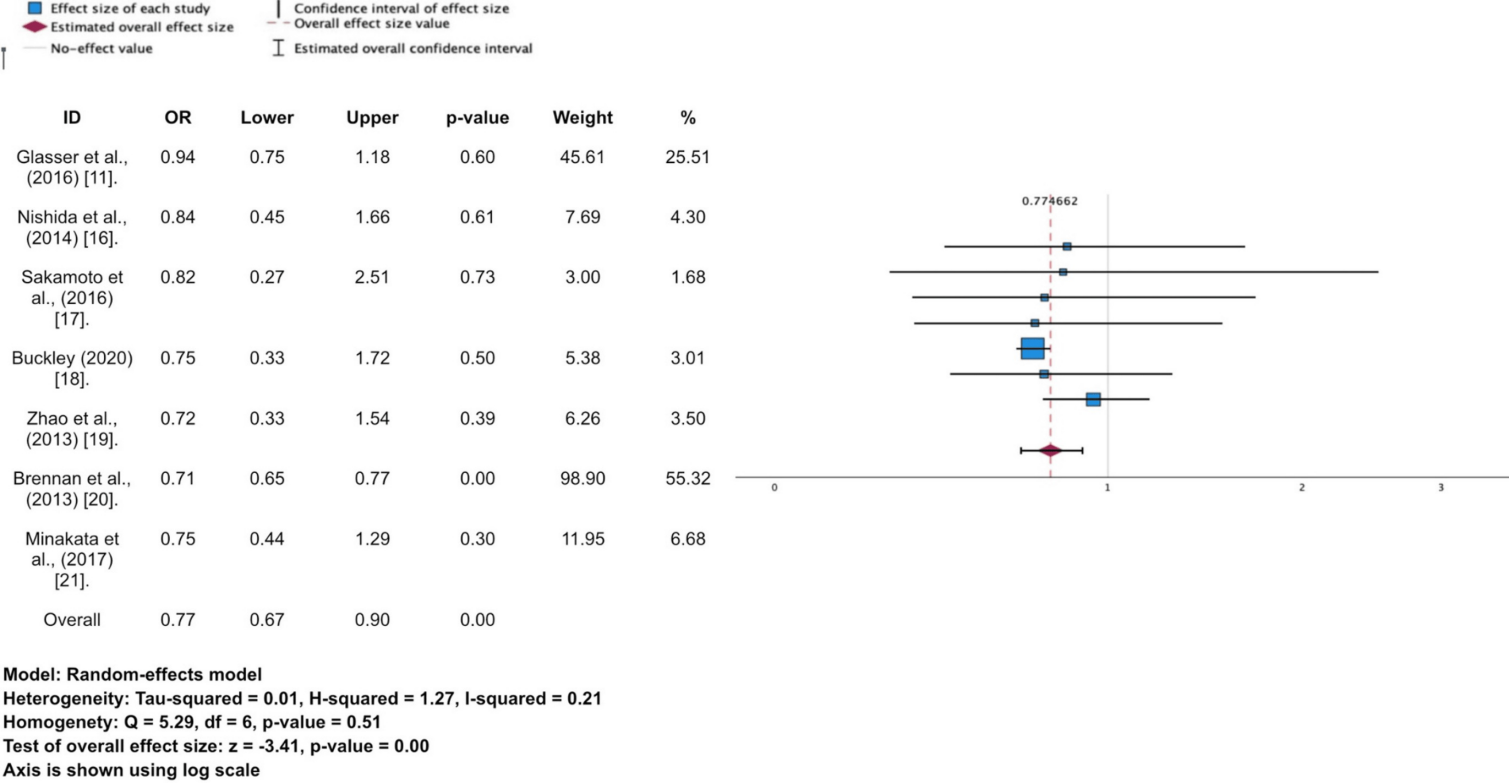
Forest plot and meta-analysis for all-cause mortality The right is favouring the mechanical valve (MV), and the left is favouring the bioprosthetic valve (BV).

Three studies reported re-operation events [16,17,21]. A total of events requiring re-operation were found in this study was 60 among 15,189 patients within the period 10 years after the surgery. Although the mechanical valve has a lower percentage in terms of the re-operation event (1.9% vs. 15.0%), the random model of meta-analysis shows that there is a significant risk difference in re-operation cases between mechanical and bioprosthetic valves (OR 0.07; 95%CI 0.03-0.17; p = 0.00) (Table 4, Figure 2). This means that there is approximately a 93% reduction of re-operation risk in 10 years among patients aged 60-70 years who receive a mechanical valve for SAVR. A heterogeneity test was performed, and it was found that there was low heterogeneity present between studies (I2 = 0%) and no homogeneity (p = 0.98). 

**Table 4:** Studies' outcomes MV: mechanical valve; BV: bioprosthetic valve

Study (n = 7)	Outcome							
	Primary outcome				Secondary outcome			
	Mortality N(%)		Re-opeation N(%)		Bleeding N(%)		Thromboembolic event N(%)	
	MV	BV	MV	BV	MV	BV	MV	BV
Glaser et al., 2016 [11]	195 (25.96)	204 (27.16)	-	-	-	-	-	-
Nishida et al., 2014 [16]	91 (71.09)	47 (74.60)	5 (3.91)	23 (36.51)	35 (27.35)	23 (36.51)	37 (28.91)	13 (20.63)
Sakamoto et al., 2016 [17]	5 (12.19)	12 (14.45)	0	12 (14.46)	4 (9.76)	4 (14.46)	2 (4.88)	2 (2.41)
Buckley et al., 2020 [18]	16 (28.57)	17 (34.69)	-	-	-	-	-	-
Zhao et al., 2023 [19]	15 (14.85)	17 (19.54)	-	-	21 (20.79)	6 (6.89)	11 (10.90)	13 (14.94)
Brennan et al., 2013 [20]	2947 (49.53)	2034 (58.03)	-	-	-	-	-	-
Minakata et al., 2017 [21]	25 (27.17)	73 (33.18)	0	20 (9.09)	8 (8.69)	24 (10.91)	8 (8.69)	0

Four studies reported major bleeding events after AVR [16,17,19,21]. A total of 125 major bleeds were found in this study among 15,189 patients within the period of 10 years after the surgery. Bioprosthetic valve has a significantly lower percentage for major bleeding (12.5% vs. 18.7%); however, the random model of meta-analysis shows that there is no significant effect of using either a mechanical or bioprosthetic valve in major bleeding events (OR 1.29; 95%CI 0.56-2.95; p = 0.55). 

**Figure 3:**
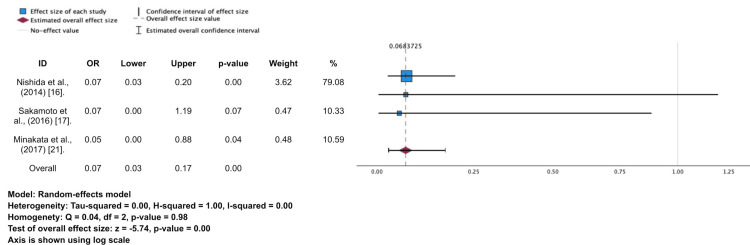
Forest plot and meta-analysis re-operation

**Figure 4:**
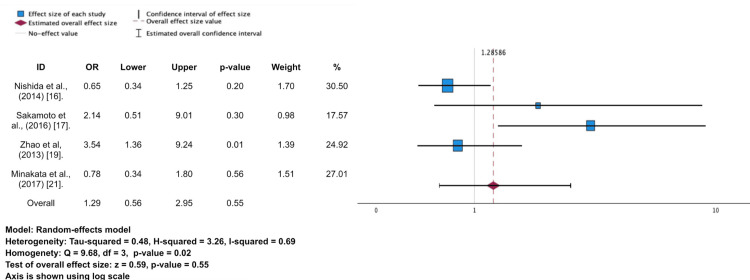
Forest plot and meta-analysis for major bleeding events

**Figure 5:**
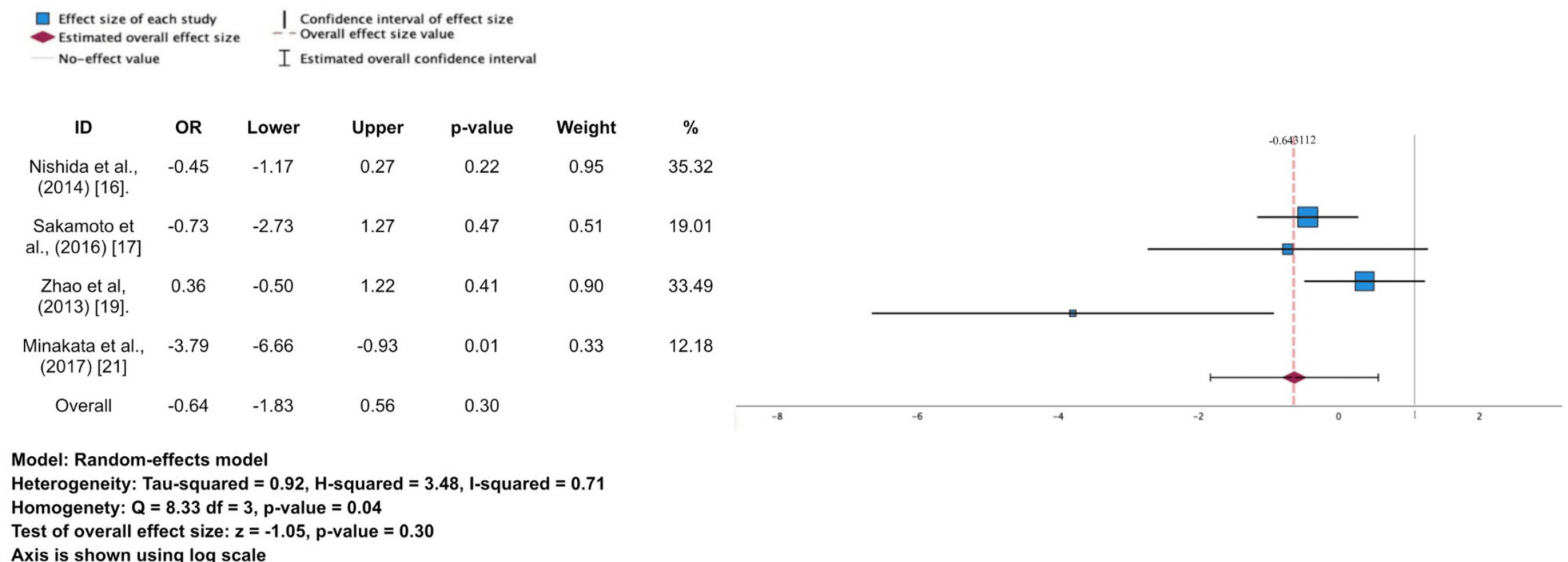
Forest plot and meta-analysis for thromboembolic events

Four studies reported thromboembolic events after AVR [16,17,19,21]. A total of 86 thromboembolic events were found in this study out of 15,189 patients within the period of 10 years after the surgery. Bioprosthetic valve has a significantly lower percentage for thromboembolic event (6% vs. 16%); nonetheless, the random model of meta-analysis shows that there was no significant effect on valve type in causing thromboembolic event (OR 0.64; 95%CI -1.83-0.56; p = 0.30). Detailed data can be seen in Table 4 and Figures 2-5.

Sensitivity Analysis and Publication Bias

In our analysis, we employed funnel plots to visually assess the potential presence of publication bias. In addition, to provide a quantitative assessment of publication bias, Egger’s weighted regression statistic was applied to all outcomes evaluated in the study. Remarkably, the results of this test yielded a p-value of 0.842 for mortality and p = 0.913 for re-operation events, indicating no discernible publication bias among the studies included in our analysis for these primary endpoints. Furthermore, when examining the secondary outcomes, including major bleeding events and thromboembolic events, Egger’s test for a regression intercept revealed no evidence of publication bias. 

Discussion

This study minimised bias and confounding by including only studies with direct comparisons between valve types, many of which used propensity score matching. Quality assessment tools (Cochrane Risk of Bias and Newcastle-Ottawa Scale) were applied, and baseline demographics showed no significant differences between groups. Statistical heterogeneity was low for primary outcomes, and no publication bias was detected. These factors strengthen the validity and reliability of our findings.

This study boasts several notable strengths. Firstly, it stands as the pioneering meta-analysis to specifically scrutinise the usage of valves in SAVR among individuals aged 60-70 years. By focusing on this specific age group, our study addresses a critical gap in the existing literature, offering valuable insights into the efficacy and outcomes of valve selection within this demographic. Secondly, our analysis revealed no substantial disparities in baseline characteristics amongst patients acquiring mechanical and bioprosthetic valves, indicating a balanced comparison and enhancing the validity of our findings. Lastly, the absence of heterogeneity in our statistical analysis further strengthens the robustness of our results. This consistency across study parameters enhances the reliability and generalisability of our findings, reinforcing the validity of our conclusions.

In this study encompassing a total of 15,189 patients, noteworthy are the findings of mortality risk reduction, wherein mechanical valves exhibited the potential to mitigate mortality events by a striking 23% over 10 years post-surgery. Moreover, the data revealed a 93% decrease in the risk of re-operation within the same timeframe, underscoring the durability and efficacy of mechanical valves in this age group. Interestingly, we found no significant association between valve type and the risk of major bleeding or thromboembolic events. This suggests that while mechanical valves may confer distinct advantages in terms of mortality and re-operation risk reduction, the valve type preferences may not exert an obvious impact on these specific complications. 

Although the most recent guidelines recommend using any valves in patients aged 60-70 years old [9,10], it is widely assumed that patients aged between 60 and 80 years tend to favor bioprosthetic valves due to a lower risk of valve dysfunction [9,11,14,16]. This inclination is evidenced by an upward trend in the utilization of bioprosthetic valves in aortic positions among patients older than 60 years, showing a remarkable increase of 78.4% since 1997 [16]. A study in the UK that used 79173 patients from the National Adult Cardiac Surgery Audit database from 1996 to 2018 shows an increase in the use of bioprosthetic valves in patients aged 60-69 years old from 24.59% to 81.87% over more than a decade [22]. It is explained that the possible increment of bioprosthetic valve usage is not only influenced by age but also by other factors such as low LVEF, the need for urgent operation, smoking, and pre-operative AF [22]. This preference stems from considerations of the weakened immune system in this age group, which may contribute to a reduced risk of re-operation-related mortality. In addition, the avoidance of lifetime anticoagulation medication associated with bioprosthetic valves potentially lowers the incidence of major bleeding accidents [23,24]. Another significant factor contributing to mortality is thromboembolic events, which discourage the use of mechanical valves due to their inherent thrombogenic characteristics [25].

Older RCTs favour a bioprosthetic valve for patients older than 60 years [9,25-27]. The oldest published RCT was the Edinburgh study that was conducted in 1975-1979, where they found no significant effect of the type of valve used in AVR on 20-year mortality and thromboembolic bleeding; however, bioprosthetic valves favour the age group due to the lower risk of major bleeding [25]. Another RCT that was done two years after was the Veterans Affairs RCT, which shows similar results to the Edinburgh study, where bioprosthetic valves show a lower risk of major bleeding events in 15 years [9]. The observed disparity in results is caused by the broad age range encompassed within the studies. Patients aged over 60 years often extend into the older than 70 age group, exhibiting distinct characteristics compared to those aged specifically between 60 and 70 years. Furthermore, an additional factor contributing to differing outcomes could be attributed to the evolution of valve technology over the past few decades. Notably, those studies utilised the Bjork-Shiley® mechanical valve, characterised by a one-leaflet tilting disc design, markedly distinct from current bi-leaflet valves. This disparity in valve structure is critical, as the Bjork-Shiley® valve exhibited significantly higher forces during disc opening, potentially leading to severe complications such as embolization, substantial regurgitation, and mortality [26]. In a slightly newer RCT from Stassano that was conducted in 1995, St Jude® and Carbomedics® mechanical valves were used in the same age population. It is found that there were no differences in risk of mortality, bleeding, and thromboembolic events by using any type of valves [27]. In this study, most studies also use the same type of bi-leaflet mechanical valve [12,16-21], for which the safety was proven through a long 25-year study, and no valve-related death was reported during the study [26].

Contrary to prior beliefs, our study reveals the superiority of mechanical valves for patients aged 60-70 years, with no notable increase in the risk of major bleeding events or thromboembolic incidents associated with bioprosthetic valves. As the first meta-analysis specifically tailored to the 60- to 70-year-old age group, it is noteworthy that parallel meta-analysis studies encompassing comparable middle-aged populations have also yielded congruent findings [28-30], thereby reinforcing the conclusions drawn from our study. A meta-analysis that was conducted in 2019, in which four propensity score-matched study and one RCT was used to evaluate valve usage in patients aged 50-70 years, found that mechanical valves have higher survival rates of 76.78% vs. 74.09% in the first 10 years compared to bioprosthetic valves. They also found a lower re-operation rate and similar bleeding and thromboembolic events in both valve types [28]. Another study published in 2022 that incorporated 13 studies shows that mechanical valve decreased 24% risk of mortality (95%CI 0.70-0.83; p < 0.001) among patients aged 50-70 years, which is similar to our study result [24]. In the same year, another meta-analysis was done in the aged group younger than 60 years, and it was found that mortality is one times higher and re-operation was three times higher in using a bioprosthetic valve; however, thromboembolic risk was similar in both valves [29]. The most recent meta-analysis study conducted in 2023 that used one RCT and 19 OS shows that in patients aged 50-70 years found no difference in early mortality and long-term cardiac death; however, mechanical valve tends to give better long-term survival and low risk of re-operation, although the bleeding risk is higher [30].

Mechanical valves are favourable due to several possible reasons. Firstly, a mechanical valve that is made out of pyrolytic carbon is known for its durability, which helps in reducing the risk of re-operation in more than 10 years, as well as lower risk of structural deterioration, which subsequently reduces the risk of valve-related mortality in older patients [23,28,31]. A study from Bourguignon et al. proved that mechanical valves have excellent long-term durability in contributing to improved survival rates in older age groups, specifically, in patients aged 60-70 years. It is found that the freedom from re-operation due to structural valve deterioration was found to be high, which was 98.1% + 0.8% in 15 years after surgery [31], which aligned with our study results. An older RCT from Stassano et al. in 2009 also found similar results, where in patients aged 55-70 years, bioprosthetic valve had more frequent valve failure and re-operation at 13 years of observation, which makes mechanical valve more favourable in that age group [27]. Current understanding suggests that modern mechanical valves have a longer durability, estimated at 20-30 years, compared to bioprosthetic valves, which typically last 10-15 years [32]. Despite its durability, activation of platelets could be triggered by high shear stress caused by blood flowing around the valves over time, which could result in thrombosis around the valve structure, increase embolism risk, and valve damage [33]. Older age is believed to be beneficial in this situation as the alteration in cardiovascular physiology among older patients could possibly affect the hemodynamic surrounding the mechanical valve, including blood flow patterns, shear stress, and turbulence within the valve prosthesis [34,35]. A study analysing alterations in aortic wall shear stress in patients aged 55-64 years found lower flow and longer deceleration phase, which is caused by lower cardiac output and vessel dilatation compared to younger groups [35]. Another study found a significant difference in peak systolic velocity and aortic wall shear stress in different age groups. Patients aged 51-60 years old have lower velocity and wall shear stress compared to younger controls [36]. Although no study using the population aged 60-70 years was found, it can be assumed that lower shear stress among older groups is impacting mechanical valve durability. Further study is needed to prove this, especially for patients aged 60-70 years. 

Secondly, the implementation of modern anticoagulation therapy in patients with mechanical valves has emerged as a promising strategy, demonstrating the potential to mitigate thromboembolic events while preserving a low risk (1-2%) of major bleeding events [23,37,38]. While it is extensively acknowledged that older patients are at an elevated risk of major haemorrhage when taking anticoagulants, the findings from this study suggest that within patients aged 60-70 years, such treatment appears to be safe. Our study also revealed no substantial difference in the risk of both major bleeding events and thromboembolic events between the two types of valves, although the included studies in this meta-analysis do not mention the type of anticoagulant that is used in their patients; therefore, the cause of the lower rate of major bleeding and thromboembolic events is still unclear. A study that analyses the effect of age on bleeding risk in anticoagulated subjects shows that in patients aged 60-69 years, the risk of major bleeding event is significantly lower than the older age group (>80 years old) with 1.8 times increased risk of bleeding in the older group [39]. Another study involving middle-aged individuals (50-70 years old) found that the risk of major haemorrhage is not significantly different compared to a younger group [40]. In another investigation assessing the risk factors associated with severe bleeding in a cohort of 232,624 patients undergoing warfarin therapy, findings revealed that individuals aged between 60 and 69 years faced a 1.67-fold increased risk of severe bleeding compared to those younger than 40 years old (95% CI 1.38-2.03). Notably, while this risk is slightly elevated compared to the 50-59 age group, it remained significantly lower than that observed in older age brackets (>70 years old). In addition, various other factors, including renal failure, liver failure, alcohol dependency, and prior bleeding events, were identified as potential contributors to the heightened risk of major bleeding events [41].

An alternative strategy to mitigate bleeding risk in patients with mechanical valves involves transitioning to safer anticoagulant options. With the advancements in medicine, there are now alternative anticoagulants available, such as novel oral anticoagulants (NOACs), which offer promising alternatives for patients requiring anticoagulation therapy. A finding from the RE-ALIGN trial shed light on the efficacy of dabigatran, an oral direct thrombin inhibitor, administered at a dosage of 300 mg twice daily. The study revealed a notable reduction in the risk of major bleeding events, with a significant 2.5-fold decrease compared to warfarin in patients with mechanical valves (95% CI, 1.23 to 4.86; p = 0.01) [35]. Despite this favourable outcome, the trial also witnessed a greater occurrence of thromboembolic events among patients taking dabigatran, albeit statistically insignificant. This phenomenon is speculated to be attributed to variations in plasma drug levels and distinct mechanisms of action compared to warfarin, prompting further exploration into alternative oral anticoagulants that can mitigate bleeding risk without compromising thromboembolic event rates [35]. Another oral anticoagulant that was tested is apixaban through the PROACT-Xa trial. This trial found a significant increase in thromboembolic event (4.2% vs. 3.4%) with no significant effect on major bleeding event [42]. In the RIWA study, which evaluated the use of rivaroxaban, a direct Factor Xa inhibitor, administered at a dosage of 15 mg twice daily, notable benefits were observed. Specifically, rivaroxaban demonstrated a reduced occurrence of thromboembolic events compared to warfarin, and notably, no major bleeding events were reported with rivaroxaban use. In addition, rivaroxaban exhibited a protective effect against minor bleeding events in comparison to warfarin, hence making it a prospective oral anticoagulant for use in modern practice [43]. However, it is important to acknowledge that the duration of follow-up in this study was limited to 90 days, which may not adequately capture the long-term effects and outcomes associated with rivaroxaban use. Further study is needed to see the long-term potential of using other anticoagulants in patients receiving mechanical valves. 

The last potential reason why mortality and re-operation risk are lower in mechanical valve is due to the lower incidence of prosthesis-patient mismatch (PPM) compared to bioprosthetic valve. PPM is defined by measuring indexed effective orifice area (iEOA) when choosing prosthesis size, where iEOA <0.85 cm^2^/m^2^ would risk PPM in the future. PPM is also known to be related to 4.6% higher mortality at 10 years after SAVR [44]. PPM may result in sustained left ventricular afterload, hindering the restoration of coronary flow reserve and impeding the reduction of left ventricular hypertrophy and dysfunction. Moreover, PPM is linked to bleeding complications, a heightened incidence of exercise-induced arrhythmias, delayed heart failure, and substantial residual regurgitation following surgery [45]. Mechanical valve offers higher iEOA values compared to bioprosthetic valve, which could affect overall mortality due to PPM [30,44-46]. The most recent meta-analysis study involving 65 published studies shows that among patients aged 60-70 years, the incidence of PPM is higher in patients receiving a bioprosthetic valve than a mechanical valve (50.9% vs. 41.5%; p < 0.001) [44]. A previous meta-analysis study utilising 34 published studies also found that using a mechanical valve could lower PPM incidence. It is also shown that mechanical valves have a larger EOA. For example, in valve size 21, St. Jude standard’s EOA is 1.4 + 0.2 and Carbomedics® is 1.5 + 0.3, which are larger than the bioprosthesis options such as Carpentier-Edwards Perimount® (1.3 + 0.4) or Mitroflow® (1.3 + 0.1) [41]. A comprehensive long-term retrospective study revealed a substantial increase in the incidence of PPM among patients aged 61-70 years compared to a younger control group (30.7% vs. 7.8%; p < 0.001). This underscores the critical importance of valve type selection in mitigating the risk of PPM. Furthermore, the study demonstrated that patients receiving mechanical valves had a notably lower overall incidence of PPM at 25.4%, contrasting starkly with the 74.6% observed among those with bioprosthetic valves. Within the cohort of PPM cases, severe PPM was notably prevalent, accounting for 23.1% among those with mechanical valves compared to a striking 76.9% among recipients of bioprosthetic valves [46].

## Conclusions

This systematic review and meta-analysis examined the efficacy of mechanical valve use in patients aged 60-70 years undergoing SAVR. While current guidelines offer flexibility in valve selection for this age group, our findings support a re-evaluation of the potential long-term benefits of mechanical valves in this population. These insights may assist clinicians in navigating the complex decision-making process surrounding valve choice in middle-aged patients undergoing SAVR.

Although our review was conducted according to the 2021 ESC guidelines, clinicians should be aware of the 2025 ESC recommendations, which provide updated guidance on prosthesis choice, timing of intervention, and patient selection. Future studies may evaluate outcomes in line with these updated recommendations. Further adequately powered randomized controlled trials specifically designed to address this age group are necessary to determine whether mechanical valves offer a definitive advantage over bioprosthetic valves in terms of long-term outcomes. Rigorous long-term follow-up, ideally spanning five to 10 years post-SAVR, is essential to ensure comprehensive data capture. At present, high-quality evidence for this critical time frame remains limited.
